# Plasma proteomics in patients with von Willebrand disease and hemophilia A highlights von Willebrand factor as main determinant of response to desmopressin treatment

**DOI:** 10.1016/j.rpth.2025.102738

**Published:** 2025-03-19

**Authors:** Jessica del Castillo Alferez, Eva R. Smit, Alexander B. Meijer, Karin Fijnvandraat, Marieke J.H.A. Kruip, Tirsa T. van Duijl, Maartje van den Biggelaar

**Affiliations:** 1 Department of Molecular Hematology, Sanquin Research, Amsterdam, Netherlands; 2 Department of Pediatric Hematology, Emma Children’s Hospital AMC, Amsterdam, Netherlands; 3 Department of Internal Medicine, Erasmus University Medical Center, Rotterdam, Netherlands

**Keywords:** DDAVP, hemophilia A, mass spectrometry, proteomics, von Willebrand Disease

## Abstract

**Background:**

Desmopressin, 1-deamino-8-D-arginin vasopressin (DDAVP), is a treatment option for people with von Willebrand disease (VWD) and hemophilia A (HA) with a large interindividual variation in response. DDAVP elicits the release of von Willebrand Factor (VWF) from endothelial cells, thereby increasing the levels of circulating VWF and coagulation factor (F)VIII. However, we currently lack detailed insight on additional systemic effects of DDAVP administration on plasma protein levels.

**Objectives:**

This study aimed to investigate plasma proteomic profiles associated with DDAVP administration.

**Methods:**

Longitudinal plasma samples of 13 patients with VWD and 9 people with mild HA up to 24 hours after DDAVP infusion were analyzed using mass spectrometry–based proteomics.

**Results:**

Among 408 proteins quantified in plasma, only VWF and VWF propeptide (pp) increased significantly at 1 and 2 hours after DDAVP infusion in people with HA and VWD, respectively. VWF antigen levels were in agreement with mass spectrometry–based VWF intensity levels (*ρ* = 0.89). A slower clearance was observed for VWF compared with that for VWFpp, accompanied with higher interindividual variation. In 4 people with HA, C-reactive protein levels increased 24 hours after DDAVP infusion, which correlated with serum amyloid A1/A2 levels.

**Conclusion:**

This study showed the selective increase of VWF and VWFpp 1 to 2 hours after DDAVP infusion and highlighted the interindividual variance in VWF clearance. Additionally, a delayed acute-phase response in a subgroup of patients suggested the potential role of inflammatory mechanisms contributing to heterogeneity of response.

## Essentials

• Desmopressin is a treatment option that increases factor VIII and von Willebrand factor (VWF) levels in plasma to manage bleeding.
• Plasma proteomic profiles were analyzed up to 24 hours postinfusion of desmopressin for systemic changes.
• Unbiased proteomics showed a selective increase of VWF and VWF propeptide 1 to 2 hours after desmopressin infusion.
• A late C-reactive protein increase suggests inflammatory responses contributing to interindividual variability.

## 1 Introduction

DDAVP (1-deamino-8-D-arginin vasopressin), desmopressin, is a safe and cost-effective prohemostatic agent that is used to prevent and treat bleeding in people with von Willebrand disease (VWD) and hemophilia A (HA) [1]. It is a synthetic analog of the hormone vasopressin that selectively binds to the vasopressin receptor (V)2 on endothelial cells, thereby eliciting the secretion of von Willebrand factor (VWF) from storage organelles called Weibel–Palade bodies (WPB) [2]. VWF is the main constituent of WPBs, where it is stored in the form of high molecular weight multimers together with its noncovalently bound propeptide (pp) and other secretory molecules, such as insulin-like growth factor binding protein (IGFBP)7 [3]. Endothelial activation results in the release of multimerized VWF into the bloodstream, where it fulfills important functions in hemostasis [4]. First, multimeric VWF serves as an adhesive molecule that supports platelet recruitment and mediates platelet aggregation at sites of vascular injury [5]. Second, VWF acts as a carrier protein for coagulation factor (F)VIII, protecting it from premature clearance from the circulation [5]. Given that DDAVP increases the plasma levels of VWF and FVIII, it is especially effective in the treatment of patients with bleeding disorders, including VWD and people with nonsevere HA [6].

DDAVP tests are recommended to evaluate efficacy of the hemostatic response before it is used to prevent and treat bleeding [7]. The mechanisms that underlie interindividual differences in DDAVP treatment efficacy are incompletely understood [7,8], and it has been hypothesized that plasma proteins besides VWF and FVIII may contribute to DDAVP efficacy, thus influencing interindividual variability of the hemostatic response [9]. To address this hypothesis, we unbiasedly studied the systemic effect of DDAVP infusion on plasma proteins using a shot-gun mass spectrometry (MS)–based approach. We demonstrate that of all detected plasma proteins (*n* = 408), only VWF and VWFpp show a rapid (<2 hours) and significant increase upon DDAVP administration, in both patients with VWD and people with HA. Extension of the monitoring period in people with HA revealed a delayed acute-phase response 24 hours after DDAVP treatment for a subset of 4 patients.

## 2 Methods

### 2.1 Patients and DDAVP testing protocol

Thirteen patients with VWD and 9 people with mild HA receiving DDAVP response testing were selected for this observational study. Patients with VWD were included upon informed consent and after approval by the Medical Ethics Committee of the Amsterdam Medical Centre (Amsterdam, Netherlands). People with nonsevere HA were enrolled between October 2011 and August 2012 in a clinical study at the Erasmus University Medical Centre (Rotterdam, Netherlands) upon approval from the Medical Ethics Committee of the Erasmus University Medical Centre, Rotterdam [10].

DDAVP (0.3 μg/kg) was administered intravenously over 30 minutes. For patients with VWD, blood was drawn before and at 2 and 4 hours after infusion, whereas for people with HA, blood was collected before and at 1, 3, 6, and 24 hours after infusion. Citrated plasma was obtained either by centrifugation at 4190*g* for 5 minutes (VWD samples) or by double centrifugation at 2000*g* and 10,000*g* for 10 minutes (HA samples). Plasma aliquots were stored at −80 °C until use.

DDAVP response was monitored by VWF antigen (VWF:Ag) for patients with VWD and by FVIII chromogenic assay (FVIII:C) for people with HA. Response was defined as complete for peak levels of FVIII:C or VWF:Ag higher than 0.50 IU/dL and as partial for peak levels of FVIII:C or VWF:Ag higher than 0.30 IU/dL and lower than 0.50 IU/dL.

### 2.2 MS-based plasma profiling

Plasma was prepared for MS analysis as previously described [11]. Briefly, 500 ng of tryptic peptides were loaded onto Evotip pure (EvoSep) tips according to manufacturer’s guidelines and separated using an Evosep One liquid chromatography system coupled to an Orbitrap Fusion Lumos Tribrid MS (ThermoFischer Scientific) equipped with an electrospray ionization source operating in positive mode using data-independent acquisition. Data were processed with DIA-NN (1.8.1) [12] by applying the library-free search with a FASTA containing reviewed proteins only (20,423 entries, downloaded on August 8, 2023). The FASTA identifier of VWF was divided into VWFpp (amino acids: 23-763) and VWF (amino acids: 764-2813), with settings described previously [11].

### 2.3 Statistical analysis

The protein group file was analyzed in the R software (version 4.2.2). Label-free quantification (LFQ) values based on 2 or more unique precursors were kept for analysis. Proteins were included in the analysis when present in at least 60% of all samples in a study (Table S1A). LFQ data were log_2_-transformed and imputed as described previously (Table S1B) [11]. Proteins were considered significant if *t*-test with Benjamini–Hochberg corrected *P*-adjusted values were <0.05 and |log_2_ fold-change| of >1, using the block function for individuals in Limma [13]. Correlation analysis based on at least 10 observations was used to evaluate the relationship of VWF LFQ to VWF:Ag (Spearman, *ρ*) and among other proteins using a correlation threshold (Pearson, *r*) of |0.7| with the Hmisc package [14]. The effect size over time for each protein was quantified using Flux [15] by calculating the area under the curve based on the median intensity at each time point normalized to the intensity at the first time point.

## 3 Results and Discussion

In total, 13 patients with VWD and 9 people with mild HA were included in this study (Table). The mean VWF:Ag response upon DDAVP testing with 0.3 μg/kg bolus injection in patients with VWD was 181 IU/dL (SD: 43 IU/dL) after 2 hours, with a mean increase of 314% (SD: 80%) compared with baseline. In people with HA, the mean FVIII:C response was 85 IU/dL (SD: 50 IU/dL) after 1 hour of DDAVP infusion, representing a mean increase of 424% (SD: 224%) compared with baseline.

**Table:** Patient selection and DDAVP testing.

Characteristic	VWD group	Hemophilia A group
Baseline		
Patient group (*n*)	13	9
VWD type 1	13	
VWD type 1	9	
Nonsevere HA		9
Age (y), mean (SD)	32 (16)	45 (14)
Sex: male, *n* (%)	6 (46)	9 (100)
VWF:Ag (IU/dL) (mean (SD)	57 (15)	128 (43)
VWF:Act (%) (mean (SD)	36 (8)	145 (59)
FVIII:C (%), mean (SD**)**	82 (17)	16 (11)
DDAVP test		
Before infusion		
VWF:Ag (IU/dL), mean (SD)	59 (16)	
VWF:Act (%), mean (SD)	42 (15)	
FVIII:C (%), mean (SD)	80 (20)	24 (16)
Peak levels^a^		
VWF:Ag (IU/dL), mean (SD)	181 (43)	
VWF:Act (%), mean (SD)	129 (42)	
FVIII:C (%), mean (SD)	328 (66)	85 (50)
Response profile^b^	0	33
Complete responders (%)	100	67
Partial responders (%)	0	33

DDAVP, 1-deamino-8-D-arginin vasopressin; HA, hemophilia A; VWD, von Willebrand disease.

a Peak levels upon DDAVP testing were determined at 2 hours in the VWD cohort and 1 hour in the HA cohort.

b A complete response was defined as an increase in VWF:Ag (IU/dL) levels to >50 in patients with VWD, or an increase in FVIII:Act(%) levels to >50 in patients with hemophilia A. Partial responders were those whose VWF:Ag levels increased to between 30 and 50 IU/dL (in VWD) or whose FVIII:Act levels rose to between of ≥30% and ≤50% (in hemophilia A).

**Figure 1:**
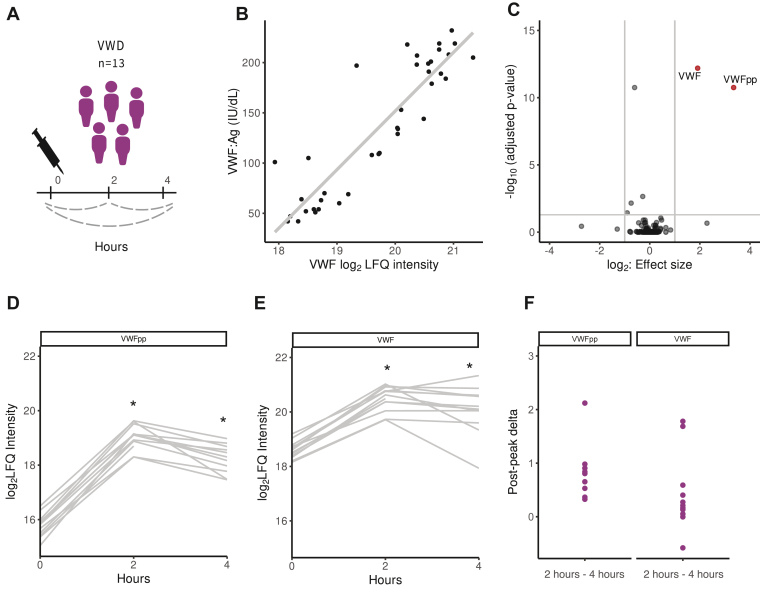
Longitudinal plasma profiling upon 1-deamino-8-D-arginin vasopressin (DDAVP) administration in von Willebrand disease **(**VWD) over 4 hours. (A) Schematic representation of blood sampling from patients with VWD (*n* = 13) after DDAVP infusion with statistical comparisons between time points annotated. (B) Comparison of von Willebrand factor (VWF) levels measured with mass spectrometry (MS) and conventional VWF:Ag (IU/dL) testing. (C) Volcano plot representing the comparison of all plasma proteins detected with MS at baseline to 2 hours after DDAVP administration. Significantly altered proteins are annotated in red (increased). Label-free quantification (LFQ) of VWFpp (D) and VWF (E) for all individuals over time after DDAVP administration. Gray lines represent individual measurements. (F) Postpeak Δ of VWFpp and VWF calculated by subtracting VWF and VWFpp intensity at time point of 4 hours from respective peak levels at time point of 2 hours. pp, propeptide. ∗Indicates significant change compared to baseline.

First, we investigated the plasma proteomic changes upon DDAVP infusion by longitudinal comparison of the protein levels in patients with VWD (Figure 1A). VWF levels determined by MS correlated with conventional VWF:Ag (*ρ* = 0.89) testing (Figure 1B), and our unbiased analysis showed, as expected, that plasma levels of VWF (1.9 log-fold, *P*_adj_ = 6.4 × 10^−13^) and VWFpp (3.3 log-fold, *P*_adj_ = 1.8 × 10^−11^) increased within 2 hours after DDAVP infusion (Figure 1C and Table S1C). Importantly, we found no additional significant protein changes 2 hours upon DDAVP infusion compared with baseline. Four hours after DDAVP infusion, VWFpp (2.1 log-fold, *P*_adj_ = 5.9 × 10^−6^) (Figure 1D) and VWF (1.5 log-fold, *P*_adj_ = 1.5 × 10^−9^) (Figure 1E) remained significantly increased. The postpeak decrease, determined by the difference in levels between peak (2 hours) and follow-up (4 hours), of VWFpp was higher compared with that of VWF (Figure 1F).

**Figure 2:**
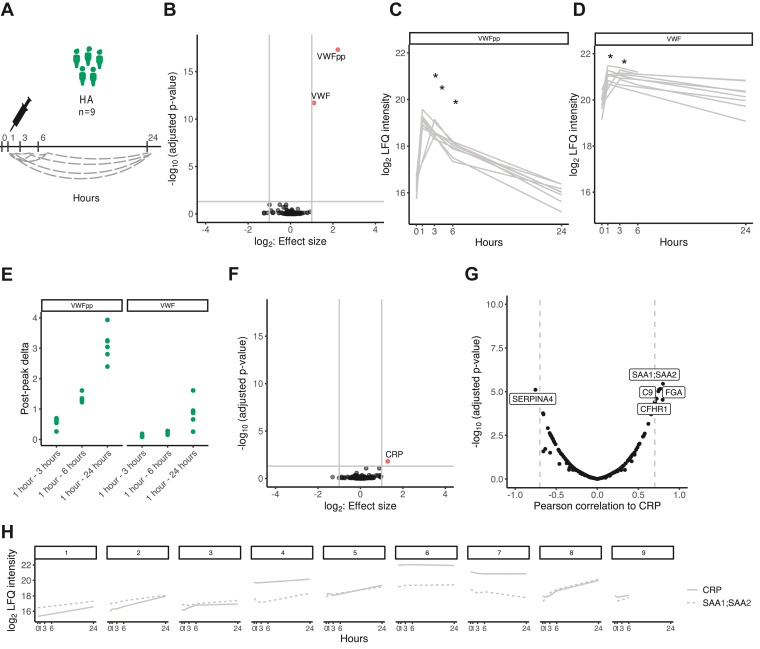
Longitudinal plasma profiling upon 1-deamino-8-D-arginin vasopressin (DDAVP) administration in people with hemophilia A (HA) over 24 hours. (A) Schematic representation of blood sampling from people with HA (*n* = 9), with statistical comparisons between time points annotated. (B) Volcano plot representing the proteome comparison at baseline to 1 hour after DDAVP administration. Significantly altered proteins are annotated in red (increased). Label-free quantification (LFQ) values of VWFpp (C) and VWF (D) for all individuals over time after DDAVP administration. Gray lines represent individual measurements. (E) Postpeak Δ of VWFpp and VWF calculated by subtracting VWF and VWFpp intensity at time points 3, 6, and 24 hours from respective peak levels (at 1 hour). (F) Volcano plot representing the comparison of 24 hours after DDAVP administration with 1 hour after DDAVP administration, with significantly altered proteins annotated in red (increased). (G) Pearson correlation of all quantified proteins to C-reactive protein (CRP) across all samples from people with HA. Proteins with an absolute correlation higher than 0.70 are labeled by gene name. (H) LFQ values of CRP and serum amyloid A-1/2 protein (SAA1; SAA2) for all people with HA over time upon DDAVP administration. Gray lines represent individual measurements. pp, propeptide; SA, serum amyloid; VWD, von Willebrand disease; VWF, von Willebrand factor. ∗Indicates significant change compared to baseline.

Next, we verified our findings of selective VWF increase in an independent selection of 9 people with HA with extended longitudinal sampling over 24 hours, which enabled the assessment of longer term postpeak clearance rates (Figure 2A). In line with patients with VWD, significant changes in plasma 1 hour after DDAVP infusion were exclusive to VWFpp (2.2 log-fold, *P*_adj_ = 1.2 × 10^−20^) and VWF (1.1 log-fold, *P*_adj_ = 1.0 × 10^−14^) (Figure 2B and Table S1D). VWFpp remained significantly increased 3 and 6 hours after infusion (Figure 2C) and VWF 3 hours after infusion (Figure 2D), underlining the increased clearance rate of VWFpp. Furthermore, the interindividual variation in postpeak differences at 24, 6, and 3 hours after DDAVP infusion when compared with peak (1 hour), were again larger for VWFpp than those for VWF (Figure 2E). This suggests that the interindividual variation in DDAVP response is influenced by differences in VWF clearance rather than VWF biosynthesis only. In addition to differences in abundance of VWF and VWFpp, a significant increase of the acute-phase protein C-reactive protein (CRP) was observed 24 hours after infusion compared with that at 1 hour after infusion (1.3 log-fold, *P*_adj__=_ .01) (Figure 2F and Table S1E). The levels of CRP correlated with acute-phase protein serum amyloid (SA)A protein isoforms 1/2 (SAA1/SAA2; *r* = 0.80), which were in line with previous observations in inflammatory response monitoring [16]. Additionally, complement factor H related 1 (*r* = 0.80), fibrinogen α chain (*r* = 0.77), complement component 9 (*r* = 0.75), and kallistatin (SERPINA4, *r* = −0.75) correlated with CRP (Figure 2G). Although significant, CRP and SAA1/2 levels were highly variable between individuals at baseline and increased more than 2-fold over 24 hours in only 4 of the 9 people with HA (Figure 2H). The observed association between VWF dynamics and the delayed inflammatory response may be related to the immunomodulatory functions of VWF. Specifically, the interaction of VWF with low density lipoprotein receptor–related protein 1 on macrophages triggers inflammatory signaling via interleukin-6 resulting in an increase of CRP [17,18]. This postulates a role for macrophage-mediated clearance of VWF, contributing to the interindividual variation of DDAVP response.

**Figure 3:**
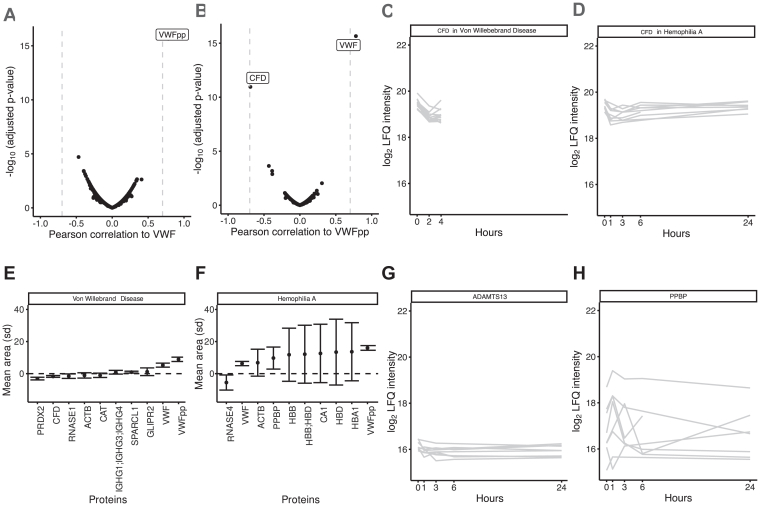
Postpeak Δ of von Willebrand factor (VWF) and VWFpp, correlation analysis and proteins of interest upon DDAVP administration in von Willebrand disease (VWD) and hemophilia A (HA) cohorts combined. Pearson correlation of all quantified proteins to VWF (A) and VWFpp (A) across all samples from both cohorts. Proteins with an absolute correlation higher than 0.70 are labeled by gene name. Label-free quantification (LFQ) values of CFD for all patients with VWD (C) and people with HA (D) over time upon DDAVP administration. Gray lines represent individual measurements. Top 10 proteins with the absolute highest mean area during the study’s time course of complete trajectories stratified for patients with VWD (*n* = 11; E) and for people with HA (*n* = 3; F), represented by the mean and SD across patients. (G, H) LFQ values of ADAMTS-13 (G) and platelet basic protein (PPBP) (H) for all individuals over time after DDAVP administration. Gray lines represent individual measurements. ADAMTS-13, a disintegrin and metalloproteinase with thrombospondin type-1 motif-13; CF, complement factor; DDAVP, 1-deamino-8-D-arginin vasopressin; pp, propeptide; SA, serum amyloid.

Since the significant response to DDAVP was limited to VWF and VWFpp, we investigated whether other plasma proteins correlated with VWF and VWFpp levels (Figure 3A, B and Table S1F). Of all quantified plasma proteins (*n* = 408), only VWFpp levels correlated with VWF (*r* = 0.79), emphasizing the exclusive VWF and VWFpp release upon DDAVP infusion (Figure 3A). For VWFpp, complement factor D levels showed an inverse correlation (*r* = −0.69), albeit just below our significance cutoff of 0.7 (Figure 3B). This transient reduction in complement factor D just after infusion (Figure 3C, D) remains elusive but could be related to endothelium activation and inflammation [19,20]. In addition to correlation analysis, we assessed the longitudinal effect size of protein levels by calculating the area of their trajectory to look for additional plasma proteins with a transient change in abundance. However, no proteins other than VWF and VWFpp showed a consistent effect size in the VWD and HA groups (Figure 3E). In the HA group, this analysis was hampered by large SDs in red blood cell–derived proteins, including hemoglobin subunits, suggesting differences from sample handling (Figure 3F) [21].

Previous studies have shown that, in addition to VWF and VWFpp, plasma levels of tissue-type plasminogen activator increase, while a disintegrin and metalloproteinase with thrombospondin type-1 motif-13 (ADAMTS-13) activity decreases in response to DDAVP administration [22,23]. Additionally, other WPB-associated proteins, including hemostatic proteins (eg, FVIII), inflammatory proteins (eg, P-selectin and interleukin 8), and proteins involved in angiogenesis (eg, IGFBP7 and angiopoietin-2) could be cosecreted with VWF [24]. Furthermore, it has been suggested that platelets become activated after DDAVP infusion and thereby contribute to a protective hemostatic effect [25]. To address these observations, we queried our data on the intraindividual coefficient of variation (CV) of these proteins across the 2 cohorts, as surrogate measure of protein changes in time. Notably, WBP constituents angiopoietin 2, tissue-type plasminogen activator, FVIII and IGFBP7 did not cross the analytical limit of detection in all samples and, thus, could not be reliably quantified using the employed approach. ADAMTS-13, however, was stable (mean intraindividual CV = 23%) in both cohorts across all time points (Figure 3G). This suggests that previously described reduction in ADAMTS-13 activity may not be related to increased clearance, but to enzymatic activity [26]. Plasma levels of typical platelet proteins, including proplatelet basic protein, platelet factor 4, thrombospondin 1 were remarkably variable (interindividual CV > 50%). Importantly, there was no indication for a general trend of the concentration of these proteins rising or decreasing following DDAVP administration (Figure 3H). This suggests that the observed variation in platelet-associated proteins may be affected by differences in the preanalytical phase of blood collection and plasma preparation [21].

Taken together, our proteomics approach revealed limited changes in plasma upon DDAVP infusion and emphasized the attribution of VWF in the direct protective hemostatic effect. The interindividual variability in DDAVP response was determined by difference in mature VWF clearance and less by peak height or time to peak height. In addition, the increase in CRP and SAA1/2 in a subset of patients indicates the delayed proinflammatory effect of DDAVP infusion. While these findings provide interesting avenues for further research, certain limitations must be considered, including the limited sample size and limited proteomic depth leaving FVIII and plasminogen activator inhibitor-1 undetected. To further assess the interindividual variation in DDAVP treatment, it would be of interest to investigate VWF clearance in relation to the VWFpp:VWF ratio and in the context of inflammation, in a larger cohort of patients, including individuals with increased clearance or a lack of response to DDAVP. To understand the mechanism underlying interindividual VWF half-life, it would be of interest to take into account its proteoforms, relevant in the interaction with FVIII, platelets, ADAMTS-13, or other WPB cargo [27]. Contributors to VWF proteoforms include posttranslation modifications, such as O-linked and N-linked glycosylation, which differ between endothelial and platelet-derived VWF [28], and common sequence variants in the D'D3, A1 and A2 region, which have previously been shown to be determinants of VWF:Ag in plasma [29].
